# Regulated intratumoral expression of IL-12 using a RheoSwitch Therapeutic System^®^ (RTS^®^) gene switch as gene therapy for the treatment of glioma

**DOI:** 10.1038/s41417-018-0019-0

**Published:** 2018-05-14

**Authors:** John A. Barrett, Hongliang Cai, John Miao, Pranay D. Khare, Paul Gonzalez, Jessica Dalsing-Hernandez, Geeta Sharma, Tim Chan, Laurence J.N Cooper, Francois Lebel

**Affiliations:** 1 Ziopharm Oncology Inc., Boston, MA USA 02129; 2 Translational Drug Development, Scottsdale, AZ USA 85259; 3 Charles River Laboratories, Worcester, MA USA 01605; 4 Intrexon Corporation, Germantown, MD 20876 USA

## Abstract

The purpose of this study was to determine if localized delivery of IL-12 encoded by a replication-incompetent adenoviral vector engineered to express IL-12 via a RheoSwitch Therapeutic System^®^ (RTS^®^) gene switch (Ad-RTS-IL-12) administered intratumorally which is inducibly controlled by the oral activator veledimex is an effective approach for glioma therapy. Mice bearing 5–10-day-old intracranial GL-261 gliomas were intratumorally administered Ad-RTS-mIL-12 in which IL-12 protein expression is tightly controlled by the activator ligand, veledimex. Local tumor viral vector levels concomitant with veledimex levels, IL-12-mRNA expression, local and systemic cytokine expression, tumor and systemic flow cytometry and overall survival were studied. Ad-RTS-mIL-12+veledimex elicited a dose-related increase in tumor IL-12 mRNA and IL-12 protein and discontinuation of veledimex resulted in a return to baseline levels. These changes correlated with local immune and antitumor responses. Veledimex crossed the blood–brain barrier in both orthotopic GL-261 mice and cynomolgus monkeys. We have demonstrated that this therapy induced localized controlled production of IL-12 which correlates with an increase in tumor-infiltrating lymphocytes (TILs) leading to the desired biologic response of tumor growth inhibition and regression. At day 85 (study termination), 65% of the animals that received veledimex at 10 or 30 mg/m^2^/day were alive and tumor free. In contrast, the median survival for the other groups were: vehicle 23 days, bevacizumab 20 days, temozolomide 33 days and anti-PD-1 37 days. These findings suggest that the controlled intratumoral production of IL-12 induces local immune cell infiltration and improved survival in glioma, thereby demonstrating that this novel regulated immunotherapeutic approach may be an effective form of therapy for glioma.

## Introduction

Glioblastoma (GBM) is the most common and aggressive primary brain tumor. These tumors are characterized by invasiveness coupled with neovascularization with a median survival of ~15 months from diagnosis through treatment with current standard of care [1, 2]. Patients with recurrent GBM have an even worse prognosis with a median survival of 6–9 months [3]. As indicated by the poor survival rate, current treatments have not been effective in preventing disease progression.

Traditionally, it was believed that the central nervous system was an immuno-privileged compartment. However, recent data have shown the presence of lymphatic drainage, increased permeability in the presence of tumor as well as antigen-presenting capability of microglial cells [4]. The GBM tumor microenvironment is associated with pathological cytokine profiles and immunosuppressive signals which prevent tumor recognition by the innate and adaptive immune system [5]. Inadequate antigen presentation in the tumor microenvironment by antigen-presenting cells (APCs) such as dendritic cells (DCs) and macrophages can lead to the development of tumor immunotolerance via T-cell exhaustion and an increase in immunosuppressive regulatory T cells (Tregs) [6].

Cancer immunotherapy attempts to harness the specificity and cytotoxic activity of the immune system to control growth and destroy tumor cells. Numerous strategies are in progress to reverse these immunosuppressive signals, including the use of immune checkpoint inhibitors which have been shown to partially reverse the immunosuppressive signals resulting in a reduction in tumor mass in some subjects [7–9]. In addition, genetically modified autologous T cells, which incorporate a chimeric gene consisting of an anti-folate single-chain antibody or modified with EGFRv3, have been shown effective in the treatment of subsets of GBM [10, 11]. Alternatively, tumor immunosuppression can be overcome by directly stimulating the immune system by the local administration of immunostimulant cytokines such as interleukin-12 (IL-12) and interferon-gamma (IFN-γ) which have been shown to be downregulated in the GBM microenvironment [12].

IL-12, a heterodimeric protein, plays a pivotal role in linking the innate and adaptive immune systems [13, 14]. IL-12 is endogenously produced by APCs and acts upon natural killer (NK) cells and T cells in the differentiation of naive CD4+ T cells to a T helper 1 (Th1) phenotype, and for activating naive T cells to activated CD8+ cytotoxic T lymphocytes [15]. Thus, IL-12 serves as a master regulator of adaptive type 1 cell-mediated immunity, a critical pathway involved in the protection against cancer. In addition to these effects, IL-12 serves as an important factor in the differentiation and survival of memory T cells [16].

Studies with recombinant IL-12 protein have been performed in multiple murine tumor models using systemically administered IL-12. Results of these studies clearly demonstrated a reduction in tumor growth rate coupled with no appreciable toxicity [17].

Based on these results, phase 1 studies were performed where recombinant IL-12 was administered systemically to human subjects. However, in the expanded phase 2 study, severe toxicity was observed and the study halted [18], thereby indicating the need to precisely control and locally administer IL-12 at the target site.

Several groups demonstrated that in vitro transduction of tumor cells with IL-12 genes could be therapeutically useful and avoid the severe toxicities observed with systemic administration [19, 20]. Direct intratumoral injection of defective recombinant adenovirus encoding IL-12 resulted in tumor regression and long-term systemic tumor immunity. However, localized administration of IL-12 by this method is not controllable, thus resulting in a narrow therapeutic index [21, 22].

The RheoSwitch Therapeutic System^®^ (RTS^®^) provides a gene expression control switch platform that confers tightly regulated, inducible gene that has been validated in clinical trials involving IL-12 [23, 24]. An RTS^®^ gene switch consists of multiple inter-dependent functional components: (1) two transcription factors, (2) an inducible promoter and (3) a small molecule activator ligand. One of the transcription factors serves as a co-activation partner, and is a fusion between a transcription activation domain and a nuclear factor domain. The second transcription factor serves as a ligand-inducible transcription factor and is a DNA binding domain fused to a nuclear factor ligand binding domain. The RheoSwitch activator ligand (veledimex) is a synthetic analog of the insect molting hormone ecdysone [25, 26]. Both fusion proteins are constitutively expressed and, in the absence of the activator ligand veledimex, provide an “off” signal with no transgene expression. In the presence of veledimex, stabilization of the heterodimeric complex between the two fusion proteins forms an active transcription factor complex, leading to transcriptional activation from the inducible promoter through recruitment of transcriptional co-activators and components of the basal transcription machinery to induce expression (“on” signal) of a gene of interest placed under the control of the RTS^®^ [26–28]. We have previously shown that Ad-RTS-mIL-12+veledimex elicited dose-related decreases in tumor growth rate with no significant change in body weight in both breast and melanoma syngeneic mouse models [25].

In this study, we explore the mechanism of action of a direct intratumoral injection of Ad-RTS-mIL-12 plus orally administered veledimex and correlate it with antitumor activity and induced systemic immunity in an orthotopic syngeneic mouse GBM model. The successful preclinical studies led to ongoing studies of Ad-RTS-hIL-12+veledimex in cancer subjects with relapsed and refractory GBM (NCT01397708).

## Materials and methods

### Cell lines and mice

A murine glioma tumor cell line, GL-261, was purchased from American Type Culture Collection (Manassas, VA). Cells were maintained in complete RPMI containing 10% heat-inactivated fetal bovine serum (Atlanta Biologicals Inc., Lawrenceville, GA). Cells were grown and maintained at 37 °C in a humidified atmosphere with 5% CO_2_. Female C57BL/6 mice, age 6–7 weeks or 20–23 weeks for the control arm of the rechallenge study, were obtained from Harlan Laboratories (Indianapolis, IN) and Charles River Laboratories (Wilmington, MA). All animal care and experimental procedures used in this study were performed in accordance with the protocol approved by the Institutional Animal Care and Use Committee guidelines.

### Adenoviral vectors and therapeutic agents

Adenoviral shuttle vectors were generated as previously described by Komita and colleagues [22] containing the mIL-12 sequences. Adenoviral vectors were generated using the RAPAd adenoviral system [23]. Ad-RTS-mIL-12 (containing and expressing the murine IL12 gene) was purified by Vivante/Lonza (Houston, TX) and stored in 20 mM Tris+10% glycerin at pH 8.2. Some preparations were also produced by Viraquest Inc. (North Liberty, IA) and stored in A195 buffer consisting of 10 mM Tris, 75 mM NaCl, 10 mM histidine, 5% sucrose (w/v), 1 mM MgC1_2_, 0.02% PS-80, 100 µM EDTA and 0.5% EtOH, pH 7.4 [24]. All preparations were negative for replication-competent adenovirus. Prior to intratumoral (IT) administration, Ad-RTS-mIL-12 was diluted into phosphate-buffered saline (PBS) to obtain the desired concentration for vector particles (vp)/injections. Virus was titrated by a plaque-forming unit assay. Bevacizumab (Avastin®, Genentech, Lot No. 557412) was supplied as a 25 mg/mL solution, and was stored at 4 °C. PD-1 inhibitor (Lot No. 5792/0915) CD279, clone RMPI-14 was received as a liquid in PBS from BioXcell Inc. (West Lebanon, NH) and was stored at 4 °C protected from light.

### Quantitative real-time PCR and RT-PCR

Mouse genomic DNA and RNA was isolated from snap-frozen tumors using the DNeasy and RNeasy nucleic acid isolation kits from Qiagen (Germantown, MD). Isolated RNA was also further treated with RNase-free DNAse (Qiagen). Quantification of isolated DNA and RNA was performed on a Nanodrop spectrophotometer (Thermo Fisher Scientific, Cambridge, MA), and further quantified using Quant-IT PicoGreen^®^ and RiboGreen^®^ assay kits from Life Technologies (Carlsbad, CA). RNA quality was also assessed using the Agilent RNA Nano kit (Agilent Technologies Inc., Lexington, MA). Absolute quantification was performed on DNA samples to determine gene copy numbers. Standard curves were generated using murine IL-12 shuttle plasmid (Intrexon Corp., Blacksburg, VA). Each reaction involved 100 ng of template DNA to determine the vector copy number and samples were run in triplicate. Real-time quantitative polymerase chain reaction (PCR) assays were performed using an ABI 7300 and/or 9300 HT system (ABI Technologies, Foster City, CA).

For relative gene expression analysis, RNA was converted to complementary DNA (cDNA) using the qScript™ cDNA SuperMix (Quanta Biosciences, Gaithersburg, MD), per manufacturer’s protocol. Equivalent amounts of cDNA were used per reaction and run in triplicate. Isolated RNA was analyzed by quantitative reverse transcription PCR (qRT-PCR; TaqMan assay) for mIL-12 and a house-keeping gene panel (mACTB). The relative expression levels of mIL-12 was based on ∆∆CT method. Assays were run using an ABI 7300 and/or 9300 HT system (ABI Technologies, Foster City, CA).

### Serum and tumor cytokine analyses

Murine IL-12-p70 (mIL-12-p70) levels were measured by enzyme-linked immunosorbent assay (ELISA) in sera and in tumor cell lysates. Frozen tumors were lysed with SDS Free Cell Culture Lysis Buffer (Promega, Madison, WI) and then pulverized with a tissue homogenizer, QIAGEN (Valencia, CA) Tissue Lyser Bead Mill, followed by 3× freeze–thaw cycles. The supernatant was removed after centrifugation at >10,000 × *g* for 5 min and used for cytokine analysis. The mIL-12p70 concentrations in the sera and tumor lysates were measured by ELISA using a Quantikine mIL-12 Immuno-assay kit (R&D Systems, Minneapolis, MN). Total protein concentration in each tumor lysate was determined using the bicinchoninic acid (BCA) method (Thermo Scientific, Waltham, MA) to calculate the pg cytokine levels /mg of tumor or /mL of sera.

### Tumor and blood flow cytometry

Brain tumors or whole blood (50 µL) samples were stained per Flow Contract Site Laboratory standard operating procedures. Briefly, samples were incubated for 30–35 min in the dark at room temperature and then washed twice with 1 mL 1× Permeabilization Buffer. The samples were resuspended in 125 µL of 1× calcium- and magnesium-free Dulbecco's phosphate-buffered saline for acquisition on the flow cytometer.

One negative sample (no antibody) was used for gating purposes. Cell populations were determined by electronic gating based on forward versus side scatter. The flow cytometer collected 20,000 CD45+ events in each tube. The CD45+ population was further characterized for T cells (CD3+/CD4+ and CD3+/CD8+), macrophages (CD11b+/Ly6G-/Ly6C-F4/80+), B cells (CD3-CD19+), NK cells (CD49b), T-cell exhaustion (LAG3+) and regulatory T cells (CD4+CD25+FoxP3+). Flow cytometric data acquisition was performed using the Facscanto ii™ flow cytometer (BD Biosciences). Data were acquired using BD FACSDiva™ software (version 8.0.1).

### Assessment of plasma, brain and cerebrospinal fluid pharmacokinetics of veledimex in mouse and cynomolgus monkey

Brain tumor, plasma and cerebrospinal fluid (CSF) samples were analyzed for veledimex using liquid chromatography/tandem mass spectrometry method. Brain tumor samples were homogenized in homogenization solution (10:90/v:v/acetonitrile:0.1% Tween 80 in water) with ratio of 1 g to 9 mL and proteins precipitated. Veledimex concentrations were calculated with a Quadratic 1/x concentration weighting linear regression over concentration ranges of 0.0500 to 50 ng/mL using deuterated (D3) veledimex as an internal standard.

### Syngeneic mouse GL-261 orthotopic glioma model

At 5 days prior to treatment initiation, female C57BL/6 (C57BL/6NHsd) mice 6–7 weeks old (Envigo; Indianapolis, IN) were inoculated intracranially with 3 µL of GL-261 murine glioblastoma tumor cells at 1 × 10^5^ cells/mouse. Implant coordinates were 1 mm anterior and 2 mm lateral to the bregma, at a depth of 3 mm. Buprenorphine (Reckitt Benckiser Healthcare; UK) was administered to the mice approximately 30 min prior to surgery. After 5 days, mice were randomized into one of the treatment groups. For those mice administered Ad-RTS-mIL-12, the vector was administered through the same bur hole at the coordinates above in a constant volume of 3–5 μL. Veledimex was administered via oral gavage once daily for 14 consecutive days.

### Data analysis

All values are expressed as the mean ± standard error of the mean. Statistical analysis was performed using a one-way analysis of variance with Dunnets post hoc test to compare differences between the groups versus control. Increased survival fractions were studied using Kaplan–Meier survival plot followed with a log-rank and Gehan–Wilcoxon tests to assess the significance of the differences. All statistical analyses were performed using GraphPrism 5 (GraphPad Software, Inc., CA, USA). Differences between groups were considered significant when *P* < 0.05. Pharmacokinetics and tissue levels of veledimex was determined using non-compartmental analysis module of the pharmacokinetic software Phoenix 64 WinNonlin.

## Results

### Veledimex crosses the blood–brain barrier in mice and cynomolgus monkeys

Veledimex was administered to separate groups of C57BL/6 mice at a single dose via oral gavage (PO) at 225 mg/m^2^. Terminal blood and CSF and brain samples were collected at 1, 2, 4, 6, 24 and 48 h post dose. Following a single oral dose of 225 mg/m^2^ veledimex, C_max_ and AUC_0-t_ in plasma were 4153 ng/mL and 4057 ng h/mL while the CSF C_max_ and AUC_0-t_ were 14 ng/mL and 466 ng h/mL. For veledimex detected in the brain, C_max_ and AUC_0-t_ were 1794 ng/mL and 25325 ng h/mL. The veledimex C_max_ and AUC_0-t_ ratio between CSF and plasma was 0.34%.

A single oral dose of veledimex at 120 mg was administered to six cynomolgus monkeys (3/gender) and plasma and CSF levels were assessed through 48 h post treatment. Since no gender-related differences were observed, the data from male and female animals were pooled. After a single oral dose of 120 mg veledimex, C_max_, and AUC_0-t_ in plasma and CSF were 327 ± 142 and 2.07 ± 0.91 ng/mL and 5887 ± 2203 and 42.5 ± 17.4 ng h/mL, respectively. The mean plasma T_max_ was observed at ~4 h post dose with elimination t_1/2_ 25.0 ± 6.1, respectively. There was ~1% of veledimex brain uptake, C_max_, and AUC_0-t_ ratios between CSF and plasma were 0.6 ± 0.2 and 0.7 ± 0.2.

In summary, these data show that orally administered veledimex crosses the blood–brain barrier in both mice and cynomolgus monkeys at sufficient levels to warrant the assessment of Ad-RTS-IL-12+veledimex in glioma.

### Demonstration of mechanism of action in vivo

A series of in vivo mechanistic studies were performed in C57BL/6 mice to demonstrated the ability of Ad-RTS-mIL-12+veledimex to produce local expression of IL-12 as well as select the optimal vector dose for further in vivo evaluation. In this study mice were administered Ad-RTS-mIL-12 1 × 10^8^ to 5 × 10^9^ vp intracranial at the coordinates stated above with veledimex administered gavage at a fixed dose of 100 mg/m^2^/day PO, and brain veledimex, viral copy number and the ability to turn on the switch to elicit local production of IL-12 mRNA and IL-12 protein expressions were assessed. The results of this study demonstrated that increasing doses of the vector in the presence of a fixed dose of activator ligand, veledimex, elicited a dose-related increase in tumor viral particles, IL-12 mRNA (activating the switch) and localized production of IL-12 (Table 1). The 5 × 10^9^ vp dose was chosen for all subsequent studies.

**Table 1:** Effects of Increasing Doses of Ad-RTS-mIL-12 Viral Particles in the Presence of a Fixed Dose of Veledimex

**Group**	**Brain veledimex C**_**max**_	**RTS gDNA**	**IL-12 mRNA**	**Tumor IL-12**
	**(ng/g)**	**(copies/100** **ng DNA)**	**(relative expression)**	**(pg/mg)**
Vehicle+vehicle		0 ± 0	1 ± 1	3 ± 1
V 100 + 1 × 10^8^ vp	515 ± 53	75 ± 50	2 ± 1	0 ± 0
V 100 + 1 × 10^9^ vp	311 ± 99	9657 ± 2641	105 ± 32	15 ± 6
V 100 + 5 × 10^9^ vp	378 ± 68	27511 ± 16109	300 ± 107	80 ± 31

*V* activator ligand, veledimex, administered orally at a fixed dose of 100 mg/m^2^/day

All data are presented as group mean ± SEM

### Correlation of veledimex dose and local cytokine production

**Fig. 1:**
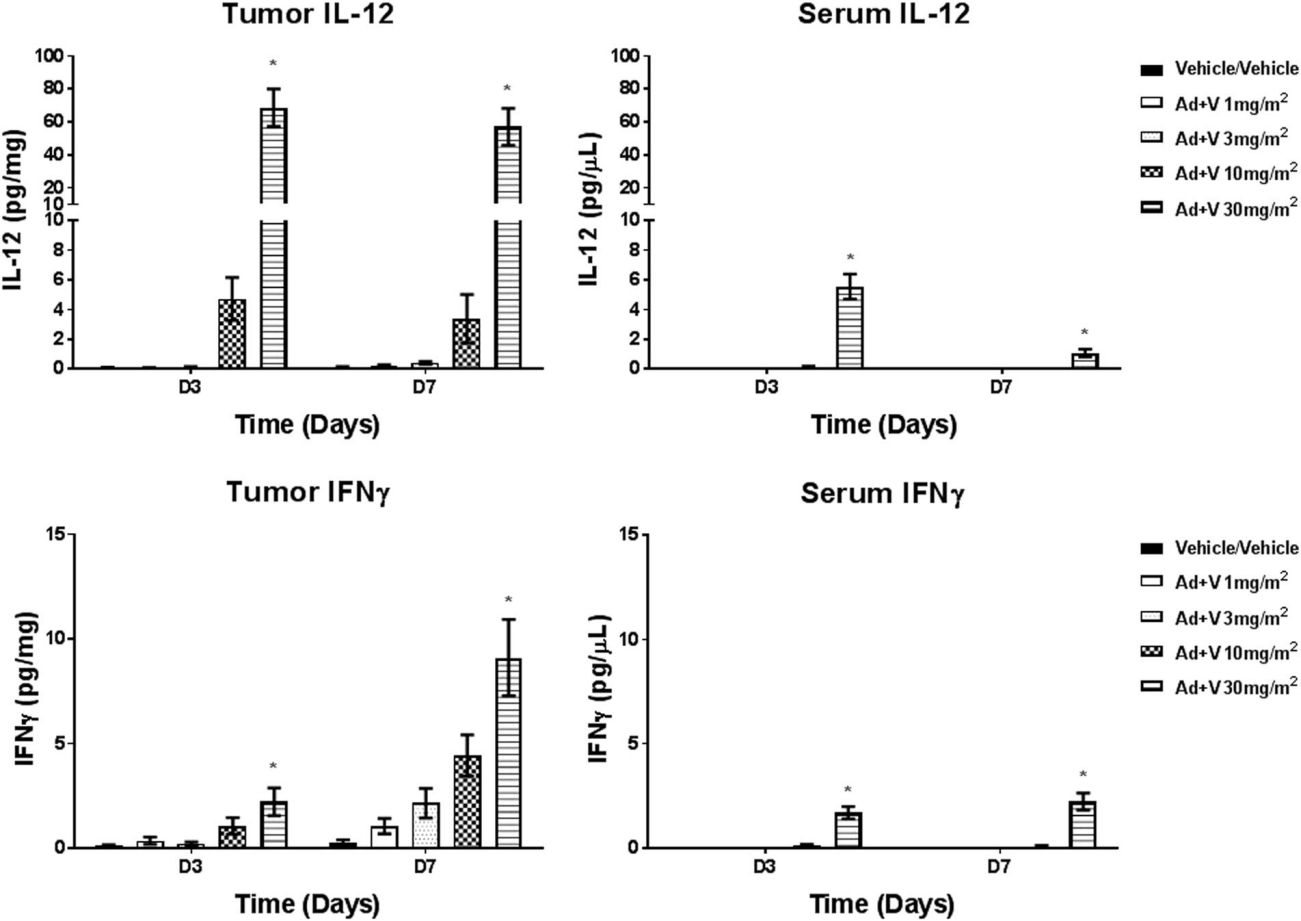
Female C57BL/6 mice were inoculated intracranially with GL-261cells. At 5 days post cell inoculation (termed as day 1 for Ad-RTS-mIL-12+veledimex treatment), mice were dosed with Ad-RTS-mIL-12 5 × 10^9^ vp intracranially and approximately 2 h later, veledimex was administered via oral gavage at 1–30 mg/m^2^/day on days 1–14. On days 3 and 7, mice in each group were killed to collect serum and tumor samples for evaluation of IL-12 and IFN-γ levels via ELISA. Each histogram depicts the mean ± standard error (*N* > 4 per time point). *P < 0.05 versus corresponding vehicle

We next explored the ability of intratumoral Ad-RTS-mIL-12 at 5 × 10^9^+oral veledimex 1 to 30 mg/m^2^ to locally produce IL-12 and downstream IFN-γ in the tumor, as well as assess serum IL-12 and IFN-γ levels in the GL-261 orthotopic syngeneic mouse model. In the tumor, there was a dose-related increase in IL-12 with steep increase in IL-12 between 3 and 10 mg/m^2^ on days 3 and 7. IFN-γ followed a similar trend with the peak increases observed on day 7 demonstrating that IL-12 produced by the vector was biologically active. Serum cytokines followed a similar trend as tumor cytokines with the levels in the serum being approximately 10 times lower than those observed in the tumor (Fig. 1).

### Ad-RTS-mIL-12+veledimex improves survival

Since the nonclinical studies demonstrated that Ad-RTS-mIL-12+veledimex was on mechanism, we initiated a series of studies to evaluate the safety and efficacy of Ad-RTS-mIL-12+veledimex in an orthotopic GL-261 glioma syngeneic mouse model. In this model, each C57BL/6 mouse received 1 × 10^5^ GL-261 glioma cells via intracranial injection ~2 mm distal to the intersection of the coronal and sagittal suture. On day 5, the animals were randomly assigned to one of the treatment groups. Animals were monitored for survival.

**Fig. 2:**
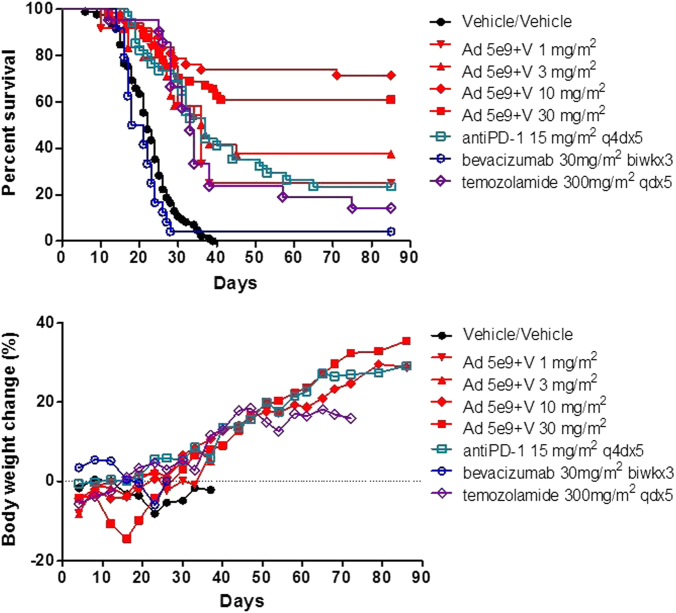
Intratumor regulated IL-12 gene delivery by Ad-RTS-mIL-12+veledimex improves survival in GL-261 glioma model. 1 × 10^5^ GL-261 glioma cells were administered into the brain. Separate groups of 12 C57BL/6 mice each were randomly assigned to one of the treatment groups. On day 1 animals were administered 1 × 10^5^ GL-261 glioma cells intracranially. On day 5, Ad-RTS-mIL-12 at 5 × 10^9^ vp+veledimex PO at 1–30 mg/m^2^/day PO was administered for 14 consecutive days and the time to disease progression and death was studied. Depicted in the upper panel are the survival results and lower panel are the respective body weights. All values are expressed as the mean for >12 animals per group. Ad-RTS-mIL-12+veledimex 10 and 30 mg/m^2^ significantly improved overall survival over all other treatment groups; *P* < 0.05

Treatment with vehicle only resulted in median survival of 23 days (Min:16, Max: 38) (Fig. 2). Consistent with disease progression, there was a marked reduction in body weight as well as increasing incidence of head tilt, ataxic locomotion, circling movements, cold to touch, emaciation, hunched posture, hypoactivity, leaning, head swelling and tremors leading to moribund killing of the animal. All vehicle-only animals were observed to have tumor present at the time of killing.

Ad-RTS-mIL-12 5 × 10^9^ vp + veledimex 1 and 3 mg/m^2^/day only slightly prolonged overall survival with the median overall survival of 36 and 27 days, respectively, when compared to vehicle. In both treatment groups, there were 3 survival animals which were terminally killed on day 85 with minimal tumor burden observed. Increasing doses of veledimex 10 and 30 mg/m^2^/day + Ad-RTS-mIL-12 5 × 10^9^ vp resulted in a significant (*P* < 0.05) sustained and prolonged survival with the median survival of greater than 85 days with ~66% of the mice surviving to terminal killing at day 85. At terminal killing, most Ad-RTS-mIL-12-treated animals were tumor free. Ad-RTS-mIL-12+veledimex 30 mg/m^2^/day elicited a moderate reduction in body weight during the veledimex dosing period which rapidly rebounded on discontinuance of veledimex (Fig. 2). In addition, these results were compared to the current and potential standards of care bevacizumab 30 mg/m^2^ biwk ×3, temozolomide 300 mg/m^2^ qdx5 and anti-PD-1 (RMP 1–4) 15 mg/m^2^ q4dx5. The anti-PD-1 and temozolomide slightly prolonged median survival to 37 and 33 days, respectively with 24 and 14% of the animals surviving to terminal killing with all mice having tumor present at terminal killing. Bevacizumab failed to prolong survival when compared to vehicle with a median survival of 20 days and 4% survival to terminal killing (Fig. 2).

### Tumor and blood flow cytometry

**Fig. 3:**
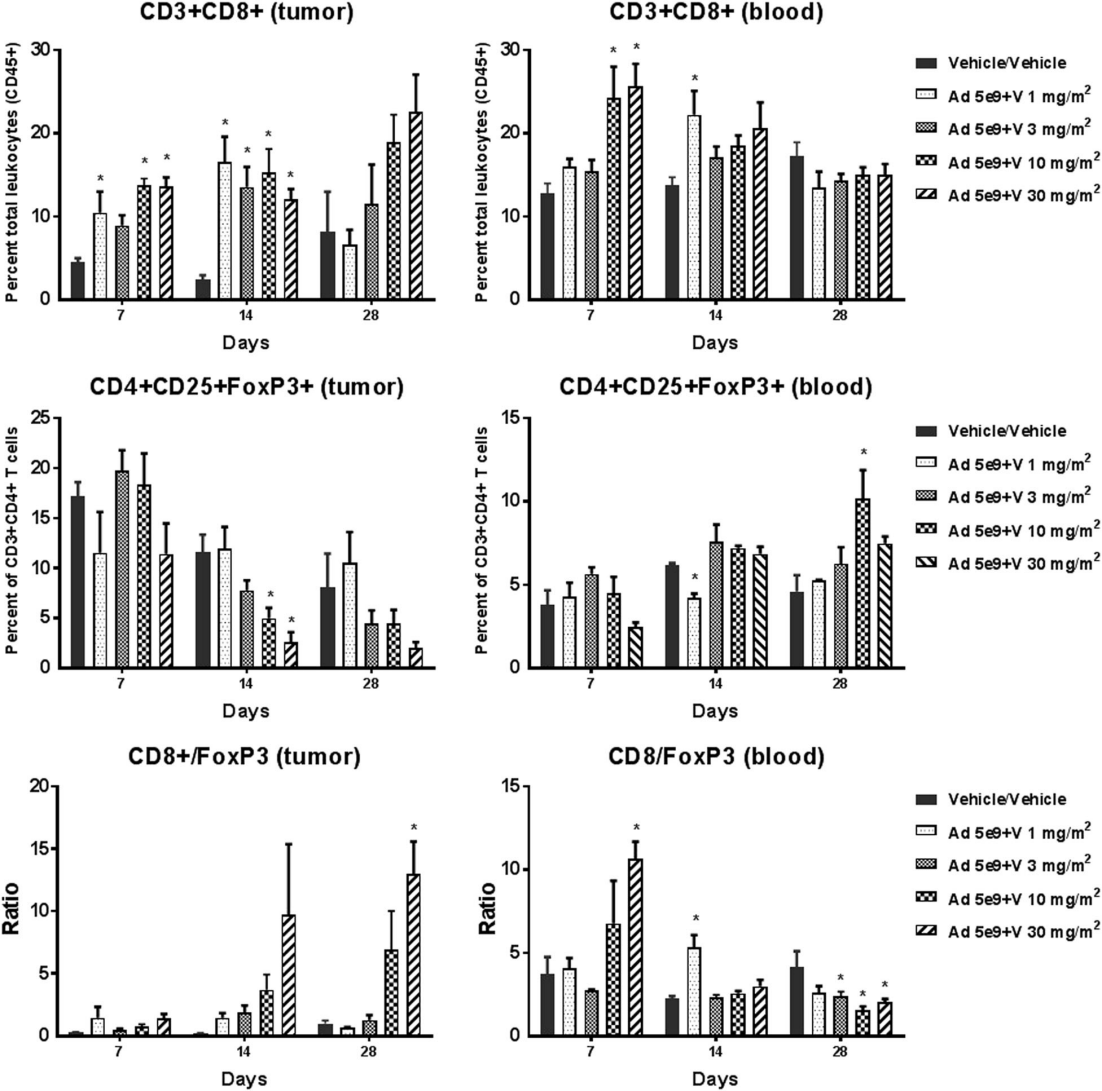
Effect of Ad-RTS-mIL-12 on tumor and blood CD8+ and FoxP3+ T cells. Mice bearing 6-day-old intracranial GL-261 tumors were administered intratumorally a single dose of Ad-RTS-IL-12 5 × 10^9^ vp+once daily orally administered veledimex for 14 consecutive days. Tumor and blood were harvested during the active dosing period and 2 weeks after the last veledimex dose. The tumor and blood were analyzed by flow cytometry for the percentage of cytotoxic T cells (CD8) and regulatory T cells (FoxP3) in the tumor (left) and blood (right). Each histogram is the mean ± SEM for 4 mice. *P < 0.05 versus vehicle on respective days

To assess the role of IL-12 on the tumor microenvironment and the recruitment of effector and regulatory T cells in the GL-261 orthotopic syngeneic mouse model, tumor and blood flow cytometry on days 7 and 14 as well as to assess persistence on day 28 (2 weeks after last veledimex dose). In the tumor at those doses which markedly prolonged survival (Ad-RTS-mIL-12+veledimex 10 and 30 mg/m^2^/day), we observed sustained increase in tumor cytotoxic T cells (CD3+CD8+) concomitant with sustained reductions in T regulatory cells (Fig. 3). There was 2.6- and 6.1-fold increase of tumor cytotoxic T cells (CD3+CD8+) in treated groups compared to vehicle group on days 7 and 14, respectively. In the efficacious dose groups, there was 2.6-fold increase of cytotoxic T cells compared to vehicle group on day 28. At the meantime, percent of Tregs (CD4+CD25+FoxP3+) was at 0.3- and 0.4-fold of vehicle level on days 14 and 28, respectively. These changes altered the tumor microenvironment in favor of cytotoxicity as demonstrated by the increase in the cytotoxic/regulatory T-cell ratio in treated groups during and after treatment. Reductions in tumor NK (CD49+) cells and increase in T-cell exhaustion (LAG3+) were also observed during the active dosing period (data not shown). In the blood Ad-RTS-mIL-12+veledimex 10 and 30 mg/m^2^/day we observed transient increases in cytotoxic T cells (CD3+CD8+) concomitant with sustained reductions in Tregs (CD4+CD25+FoxP3+) on day 7 (Fig. 3). There was an ~ 2-fold increase in cytotoxic T cells as well as cytotoxic T cells/Treg ratio in efficacious dose groups on day 7. No clear trend was observed in blood samples on days 14 and 28. The above data indicated that T-cell changes were more robust in tumor microenvironment than in the whole blood. (Fig. 3).

### Demonstration of improved survival upon rechallenge

**Fig. 4:**
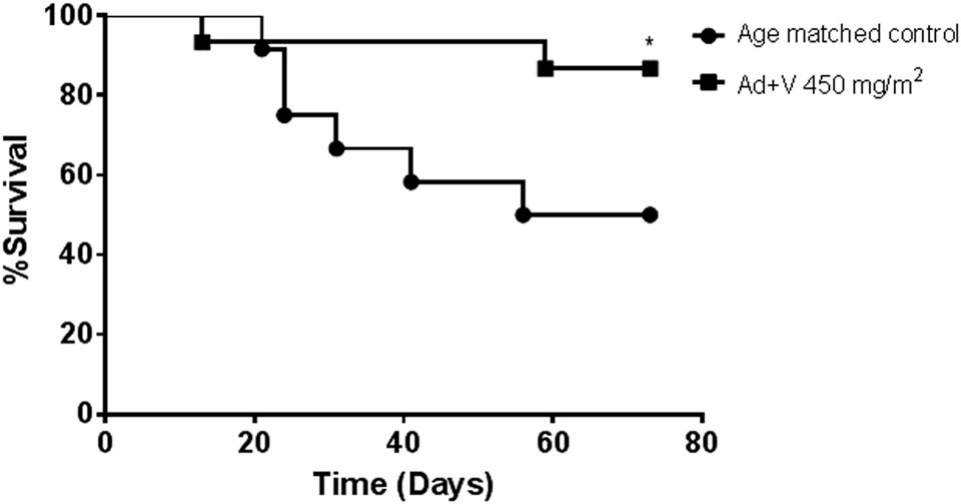
Intratumor regulated IL-12 gene delivery by Ad-RTS-mIL-12+veledimex induces systemic immune memory in the GL-261 orthotopic mouse glioma model. Surviving animals that had been previously treated with Ad-RTS-mIL-12+veledimex 450 mg/m^2^/day were rechallenged with 1 **×** 10^5^ GL-261 glioma cells at the same coordinates as the prior studies (*N* = 36). The control group consisted of 12 age-matched C57BL/6 mice (20–23 weeks old) inoculated with 1 × 10^5^ GL-261 glioma cells and survival monitored for an additional 73 days. *P < 0.05 versus age-matched control

Prior treatment with Ad-RTS-mIL-12+veledimex produced a durable survival response that was superior to standards of care and vehicle controls. To determine if pretreatment with Ad-RTS-mIL-12+veledimex provides sustained benefit upon tumor rechallenge, on day 1, 36 surviving animals previously treated with Ad-RTS-mIL-12 (1 × 10^10^ vp)+veledimex (450 mg/m^2^/day PO) were rechallenged with 1 × 10^5^ GL-261 cells at the site of original implantation. In addition, 12 age-matched (20–23 weeks) C57BL/6 mice were administered intracranial 1 × 10^5^ GL-261 cells and survival monitored for an additional 73 days. The results of this study clearly show that Ad-RTS-mIL-12+veledimex demonstrated a significant increase in survival with ~90% of the animals surviving until the end of post rechallenge monitoring period vs. 50% for naive age-matched control group (*P* < 0.05) (Fig. 4).

## Discussion

These studies provide proof of concept that regulated and controlled local IL-12 production can overcome the innate immunosuppression in glioma resulting in tipping the balance via increasing tumor cytotoxic T cells and reducing tumor Treg cells resulting in prolonged survival in an orthotopic model of glioma.

We chose the GL-261 orthotopic model since it closely mimics the human GBM phenotype in that it has K-ras oncogene as well as mutant p53 suppressor gene concomitant with high levels of c-myc [29–32]. In addition, the GL-261 tumor is partially immunogeneic as demonstrated by the presence of major histocompatibility complex I with low levels of costimulatory molecules required for T-cell activation, i.e., MCHII, B7–1 and B7-2 [29]. Tumors formed from GL-261 cells mimic the same four stages of growth over a 4-week period that is observed in human GBM. Following implantation, there is perivascular organization followed by proliferation, hypoxia, neovascularization and development of necrotic regions. The GL-261 tumors, while invasive, do not metastasize outside the brain and do not spontaneously regress [29, 33].

IL-12 is a potent immunostimulatory cytokine extensively investigated in cancer therapy, and has been shown to increase survival in rodent glioma models [34–36]. IL-12 facilitates the development of an inflammatory IFN-γ- secreting tumor-specific Th1-type immune response, thereby enhancing tumor cytotoxicity.

Systemic administration of recombinant IL-12 protein in mice has demonstrated a reduction in tumor growth rate coupled with no appreciable toxicity. [17]. However, in phase 1 studies systemic administration of recombinant IL-12 resulted in severe toxicity [18], thus demonstrating the need to precisely control and locally administer IL-12 at the target site. To assess the ability of localized IL-12 to have an acceptable therapeutic index, Wei et al. [37] used lentiviral vectors to transduce SCCVII tumor cells to produce local IL-12 at different concentrations. Their results demonstrated that the local delivery of IL-12 is an effective route for overcoming innate tumor immunosuppression. To assess the role of local IL-12 administration in a GL-261 orthotopic model of GBM, Vom Berg et al. [38] implanted osmotic pumps to ensure continuous dosing of IL-12 to the tumor. They found that local IL-12 administration reversed the GBM-induced immunosuppression, leading to increase in overall survival. While both approaches demonstrated the ability of local IL-12 to overcome innate tumor immunosuppression, both approaches are cumbersome and are difficult to precisely control local IL-12 levels in the clinical setting. Intratumoral delivery of DCs allows the capture and presentation of tumor antigens, and DCs have been shown to cause tumor regression in a breast cancer in vivo model, and prolonged survival and immunity to tumor rechallenge in rats implanted with glioblastoma cells [39, 34]. With this in mind, Komita et al. [26] studied a conditional IL-12 expression system that is tightly controlled by an orally administered activator ligand. In their study, they transduced DC cells with Ad-RTS-mIL-12 and administered the cells into a B16 melanoma tumor. They observed that the administration of the activator ligand resulted in controlled local production of IL-12 leading to an increase in cytotoxic T cells and tumor regression.

In the present study, we assessed an adenoviral vector that expresses IL-12, i.e., Ad-RTS-mIL-12 or Ad-RTS-hIL-12 under the control of a conditional (regulated) promoter, administered together with an orally bioavailable small molecule activator ligand, veledimex. The RTS^®^ “gene switch” functions as a conditional (regulated) promoter of transgene expression which can be controlled by a small molecule ligand. RTS-inducible transgene expression is “off” in the absence of veledimex, whereas transgene expression is turned “on” by the administration of veledimex. In the GL-261 orthotopic model of glioma, we demonstrated a dose-related increase in tumor IL-12 and downstream IFN-γ, demonstrating that the IL-12 produced was biologically active.

Successful immunotherapy involves overcoming the immunosuppressive tumor environment as demonstrated by low levels of cytotoxic T cells and increased Tregs. We have shown that Ad-RTS-mIL-12+veledimex elicited a dose-related increase in tumor IL-12 which in turn elicited a dose-related increase in CD3+CD8+cytotoxic T cells concomitant with a reduction in FOXP3+ regulatory T cells. On day 14, cytotoxic T cells were increased by ~ 6-fold over vehicle and at the efficacious doses of veledimex 10 and 30 mg/m^2^. The increase persisted for at least 14 days after the last dose of veledimex. Concomitant with the increase in cytotoxic T cells there was a marked decrease in Tregs, thus demonstrating that Ad-RTS-mIL-12+veledimex could stimulate and restore local immune function in a dose-related manner. Numerous investigators have also demonstrated that tumor-infiltrating T cells and more importantly the ratio of cytotoxic T cells/Tregs provide a favorable prognostic marker to predict the success of immunotherapies in breast [40], ovarian [41], melanoma [42] and glioma [43–45]. In the present study, we also observed transient increases in the blood CD3+ CD8+/FoxP3 ratio which may be useful as a surrogate marker for efficacy. However, further studies are required to establish its role as a surrogate marker for efficacy.

The local increase in IL-12 increase coupled with the local restoration of the immune system resulted in a concomitant increase in long-term survival without significant adverse events. At day 85 (study termination), over 95% of the Ad-RTS-IL-12+veledimex-treated animals were tumor free. In contrast, bevacizumab, temozolomide and anti-PD-1 therapy only slightly prolonged survival. Similar survival benefit was observed by others [44–46]. To assess the ability of Ad-RTS-IL-12+veledimex to provide sustained benefit on rechallenge, a group of surviving animals were readministered 1 × 10^5^ GL-261 tumor cells 100 days after Ad-RTS-IL-12+veledimex. We observed ~90% of the animals surviving until the end of the 73-day post-rechallenge monitoring period vs. 50% for the naive control group, thus demonstrating the induction of local immune response induced by vector intratumoral administration and oral veledimex.

In summary, our results demonstrate that the localized delivery of IL-12 encoded by the replication-incompetent adenoviral vector Ad-RTS-IL-12, and controlled by the oral activator veledimex is an effective gene and immunotherapeutic strategy in preclinical studies. We have demonstrated that this therapy induced localized controlled production of IL-12 which correlates with an increase in tumor-infiltrating lymphocytes leading to the desired biologic response of tumor growth inhibition and regression. These results demonstrate the need to study Ad-RTS-IL-12+veledimex in the patients with glioma. Indeed, clinical trials based on these data have been initiated in the treatment of glioma (NCT02026271) via direct intratumoral injection of adenoviral particles carrying a gene switch and the human IL-12-p70 transgene and oral administration of veledimex to produce hIL-12. The preliminary results of this study are encouraging [47]. Thus, the local tightly controlled production of IL-12 has the potential to significantly expand the cancer immunotherapeutic armamentarium.
